# Preoperative overnight parenteral nutrition (TPN) improves skeletal muscle protein metabolism indicated by microarray algorithm analyses in a randomized trial

**DOI:** 10.14814/phy2.12789

**Published:** 2016-06-07

**Authors:** Britt‐Marie Iresjö, Cecilia Engström, Kent Lundholm

**Affiliations:** ^1^ Department of SurgeryInstitute of Clinical SciencesSahlgrenska AcademyUniversity of GothenburgGöteborgSweden

**Keywords:** Cell signaling, mTOR, parenteral nutrition, protein synthesis, skeletal muscle

## Abstract

Loss of muscle mass is associated with increased risk of morbidity and mortality in hospitalized patients. Uncertainties of treatment efficiency by short‐term artificial nutrition remain, specifically improvement of protein balance in skeletal muscles. In this study, algorithmic microarray analysis was applied to map cellular changes related to muscle protein metabolism in human skeletal muscle tissue during provision of overnight preoperative total parenteral nutrition (TPN). Twenty‐two patients (11/group) scheduled for upper GI surgery due to malignant or benign disease received a continuous peripheral all‐in‐one TPN infusion (30 kcal/kg/day, 0.16 gN/kg/day) or saline infusion for 12 h prior operation. Biopsies from the rectus abdominis muscle were taken at the start of operation for isolation of muscle RNA. RNA expression microarray analyses were performed with Agilent Sureprint G3, 8 × 60K arrays using one‐color labeling. 447 mRNAs were differently expressed between study and control patients (*P* < 0.1). mRNAs related to ribosomal biogenesis, mRNA processing, and translation were upregulated during overnight nutrition; particularly anabolic signaling S6K1 (*P* < 0.01–0.1). Transcripts of genes associated with lysosomal degradation showed consistently lower expression during TPN while mRNAs for ubiquitin‐mediated degradation of proteins as well as transcripts related to intracellular signaling pathways, PI3 kinase/MAPkinase, were either increased or decreased. In conclusion, muscle mRNA alterations during overnight standard TPN infusions at constant rate altered mRNAs associated with mTOR signaling; increased initiation of protein translation; and suppressed autophagy/lysosomal degradation of proteins. This indicates that overnight preoperative parenteral nutrition is effective to promote muscle protein metabolism.

## Introduction

Malnutrition is associated with increased morbidity and mortality in hospitalized patients suffering from either progressive disease or scheduled for interventional treatments such as surgery and neoadjuvant oncological treatment (Bosaeus et al. 2002; Kiss et al. 2014). Such facts have supported the view that artificial nutrition, by nasogastric tube feeding and total parenteral nutrition should improve outcome, which has been challenged by numerous randomized and prospective investigations (Drott et al. 1988; Sandstrom et al. 1995; Harrison et al. 1997; Lundholm et al. 2004; Takesue et al. 2015). However, outcome results have not been unanimously clear cut, although some studies have supported beneficial effects by nutritional support during disease progression and supportive modality to surgery (Lundholm et al. 2004; Dillon et al. 2007). Thus, modest effects by short‐term nutrition have raised the question to what extent institution of early perioperative nutrition may really induce activation of protein synthesis toward protein balance, particularly in rather inactive skeletal muscles (Tjader et al. 1996). This uncertainty has remained despite application of sophisticated research methodology to demonstrate net flux of amino acids into defined protein pools and tissue compartments, in part due to method limitations such as difficulties to define the immediate precursor pool of labeled amino acids as markers of initiation of protein translation (Davis and Reeds 2001; Iresjo et al. 2005; Chow et al. 2006; Tran et al. 2015). This dilemma has conserved a scientific discussions whether protein synthesis or protein degradation of muscle proteins are most important alterations when short‐term intravenous feeding is provided (Sandstrom et al. 1995; Tjader et al. 1996; Chow et al. 2006). This uncertainty has led to nihilism among physicians concerning the impact of nutritional support to a majority of cancer patients among others. Therefore, the aim of this study was to confirm evidence that standard overnight preoperative TPN to patients, at risk for undernutrition following major surgery, promotes anabolism measured by algorithmic microarray analyses according to our reported findings of increased S6K1 phosporylation and increased eIF4E‐eIG4G protein complex formation during overnight TPN infusion (Iresjo et al. 2008).

## Methods

### Patients, blood, and tissue sampling

A total of 22 patients who underwent upper gastrointestinal surgery at Sahlgrenska University hospital due to malignant or benign disease were included in the study, based on their potential need for postoperative artificial nutrition according to risks on group level for patients who undergo major surgery (Table**** 1). Patients were diagnosed with pancreatic‐ (TPN/Saline, *n* = 2/3), gastric‐ (*n* = 4/2), esophageal carcinoma (*n* = 3/2) cholangiocarcinoma (*n* = 1/1), small bowel (*n* = 0/1), or benign gastrointestinal disorder (*n* = 1/2). Exclusion criteria were insulin‐dependent diabetes or steroid medication. The day before operation, patients were randomized to receive either a continuous overnight infusion of total parenteral nutrition (TPN/11 patients) or physiological saline (controls/11 patients). Randomization was done after the patient was recruited to the study by the responsible physician. Randomization was done by a computer algorithm based on age, sex, cancer (type of cancer)/non ca, height, weight, % weight loss (compared to predisease weight). Patient data for the randomization were collected by a study nurse and registered in the data program. The responsible physician had no impact over which treatment the patient received. Due to analytical reasons, no other blinding of samples was done. Data from all included patients were obtained. The nutrition was provided as all in one infusions (Kabiven Perifer^®^, Fresenius Kabi AB, Uppsala, Sweden) at a rate providing 30 kcal/kg/day and 0.16 gN/kg/day. Infusions started at 6 pm and lasted at constant rate until muscle biopsies were taken next morning at the start of surgery (12–16 h later). Muscle biopsies from the rectus abdominis muscles were taken as the first procedure at the start of operation and immediately placed in RNA later solution (Ambion, Life Technologies, Europe BV, Bleiswijk, the Netherlands). Samples were kept at +4°C for 24 h and thereafter frozen at −20°C until analyses. Biopsies were removed using a minimum of handling. Blood samples from arteria radialis were collected at the same time as muscle biopsies for the measurement of amino acids and substrates in serum or plasma. Patient characteristics are shown in Table 1. Data on translation initiation factor complexes for protein synthesis and mRNA expression of myosin transcripts have been reported in previous publications which included 10 of the present 22 patients (Iresjo et al. 2008; Iresjo and Lundholm 2012). Biopsies were collected in year 2005–2006 (10 patients) and year 2011–2012. Ethical approval for the study was obtained from the regional board of ethics in Gothenburg.

**Table 1:** Patient characteristics when randomized to receive overnight preoperative TPN or saline infusion. Blood samples were taken before start of infusions (mean ± SE)

	Saline infusion	TPN infusion	*P*
Age (years)	68 ± 2	69 ± 3	0.81
Male/Female (*n*)	6/5	6/5	ns
Cancer/noncancer (*n*)	9/2	10/1	ns
BMI	23.8 ± 1.3	24.5 ± 1.1	0.68
Weight (kg)	68 ± 4	73 ± 4	0.39
Weight loss (%)	6.8 ± 3.0	6.2 ± 1.7	0.86
Hb (g/L)	132 ± 5	140 ± 4	0.20
Sodium (mmol/L)	139 ± 1	140 ± 1	0.44
Potassium (mmol/L)	4.1 ± 0.1	4.2 ± 0.1	0.56
Calcium (mmol/L)	2.39 ± 0.04	2.39 ± 0.02	0.99
Protein (g/L)	62 ± 6	70 ± 2	0.26
Creatinine *μ*mol/L)	66 ± 5	72 ± 5	0.41
ALP (*μ*mol/L)	2.1 ± 0.3	1.7 ± 0.3	0.40
ASAT (*μ*mol/L)	0.38 ± 0,02	0.47 ± 0.05	0.19
ALAT (*μ*mol/L)	0.35 ± 0.05	0.44 ± 0.08	0.36
Bilirubine (*μ*mol/L),	18 ± 6	15 ± 6	0.71

ASAT, aspartate aminotransferase; ALAT, alanine aminotransferase; ALP, alkaline phosphatase; ns, not significant (anova and *χ* ^2^ testing).

### Microarray analysis

Muscle biopsies were homogenized using a rotor‐stator homogenizer. RNA was extracted with RNeasy fibrous tissue kit with DNase step included according to manufacturer's instructions (Qiagen AB, Sollentuna, Sweden). RNA concentration and purity were checked by spectrophotometric measurement using a Nanodrop ND‐1000 instrument and RNA quality were determined using Agilent Bioanalyzer 2100 with RNA 6000 nano kit (Agilent Technologies, Waldbronn, Germany). RNA integrity number was above 8.5 for all samples. Two hundred nanograms of RNA from each sample was labeled with Cy3 in a cDNA synthesis reaction following the “Low input quick amp labeling protocol G4140‐90040 version 6.5” (Agilent Technologies, Germany).Cy3 labeled cDNA from the samples were then hybridized on 8x60K Agilent SurePrint G3 v2 Human Gene Expression Microarrays, during 17 h followed by posthybridization washes. Microarrays were scanned for quantification of signals in an Agilent Microarray Scanner G2505C and preprocessed with Feature extraction software program version 10.7.3.1. Feature extraction files were imported into Genespring GX 13.1.1 software (Agilent Technologies, Santa Clara, CA) for further analysis. Files were filtered on flags to remove outlier and background expressed features (signal less than 2.6 SD above background signal) before normalizing to the 75th percentile. 26109 of the 50739 transcript features on the array were indicated as detected after filtering on flags. Based on the appearance of the volcano plot, features with less than 1.2‐fold change were removed before statistical evaluation (5500 features remaining) which was done by *t*‐test with Benjamini–Hochberg correction for multiple significance. Further details on data analyses are described in the results section.

### Blood analyses

Measurements of substrate levels (glucose, insulin, glycerol, free fatty acids, triglycerides) were performed as standard analyses at the Clinical Chemistry Laboratory at Sahlgrenska University Hospital. Quantification of amino acid concentrations was performed as described elsewhere (Iresjo et al. 2008).

### Statistics

Results are presented as mean ± SE. Patient characteristics and blood tests were compared between groups by analysis of variance (ANOVA). *P* ≤ 0.05 was regarded statistically significant. mRNA alterations were compared between groups by *t*‐test with Benjamini–Hochberg correction for multiple significance. mRNA transcripts were selected at *P* ≤ 0.10 for subsequent algorithm analyses, which were regarded statistically significant at *P* ≤ 0.05. All individual mRNA transcripts reported to be altered in Result and Discussion sections had *P* ≤ 0.1.

## Results

### Substrates and amino acids in arterial blood

Insulin, free fatty acids, and triglyceride concentrations increased significantly during TPN infusion (*P* < 0.05), whereas glucose and glycerol levels were not significantly different in TPN‐infused patients compared to saline‐infused control patients (Table**** 2).

**Table 2:** Substrate levels in arterial blood plasma or serum after overnight infusion of TPN or saline at constant rate (mean ± SE)

	Saline infusion	TPN infusion	*P* ≤
p‐Glucose (mmol/L)	7.1 ± 0.5	8.7 ± 0.6	ns
s‐Insulin (mU/L)	9 ± 5	28 ± 7	0.05
s‐Glycerol (mmol/L)	0.14 ± 0.03	0.20 ± 0.02	ns
p‐FFA (mmol/L)	0.71 ± 0.10	0.46 ± 0.05	0.05
s‐Triglycerides (mmol/L)	1.27 ± 0.16	2.12 ± 0.2	0.01

ns, not significant.

Total plasma amino acid concentrations as well as total essential amino acid concentrations increased significantly during TPN infusion compared to saline infusion. Valine and isoleucine increased among the BCAA while leucine was not changed. Methionine, phenylalanine, and threonine increased among the essential amino acids, whereas lysine and tryptophan were unchanged. Five of the thirteen measured nonessential amino acids increased significantly (Ala, Arg, Asp, Gly, His) (Table**** 3).

**Table 3:** Amino acids in arterial blood plasma after overnight infusion of TPN or saline at constant rate (mean ± SE)

AA *μ*mol/L	Saline infusion	TPN infusion	*P*≤
Essential amino acids			
Isoleucine	66 ± 6	98 ± 6	0.01
Leucine	124 ± 12	132 ± 9	ns
Valine	229 ± 22	323 ± 19	0.01
Total BCAAs	419 ± 39	553 ± 33	0.05
Lysine	171 ± 18	191 ± 16	ns
Methionine	28 ± 3	69 ± 4	0.001
Phenylalanine	53 ± 3	97 ± 6	0.001
Threonine	121 ± 11	177 ± 11	0.01
Tryptophan	20 ± 4	29 ± 5	ns
Total essential AAs	812 ± 69	1118 ± 57	0.01
Nonessential amino acids			
Alanine	361 ± 51	539 ± 36	0.01
Arginine	79 ± 12	129 ± 12	0.01
Aspartic acid	5 ± 1	13 ± 2	0.01
Citrulline	25 ± 4	28 ± 5	ns
Glutamic acid	109 ± 9	132 ± 14	ns
Glutamine	538 ± 49	540 ± 35	ns
Glycine	233 ± 18	368 ± 32	0.01
Histidine	73 ± 6	115 ± 6	0.001
Serine	124 ± 9	147 ± 10	ns
Taurine	61 ± 12	61 ± 7	ns
Tyrosine	63 ± 6	46 ± 3	ns
Ornithine	63 ± 6	83 ± 9	ns
* α*‐aba	18 ± 4	25 ± 3	ns
Total amino acids	2564 ± 205	3347 ± 174	0.01

ns, not significant.

### Microarryanalysis

**Figure 1:**
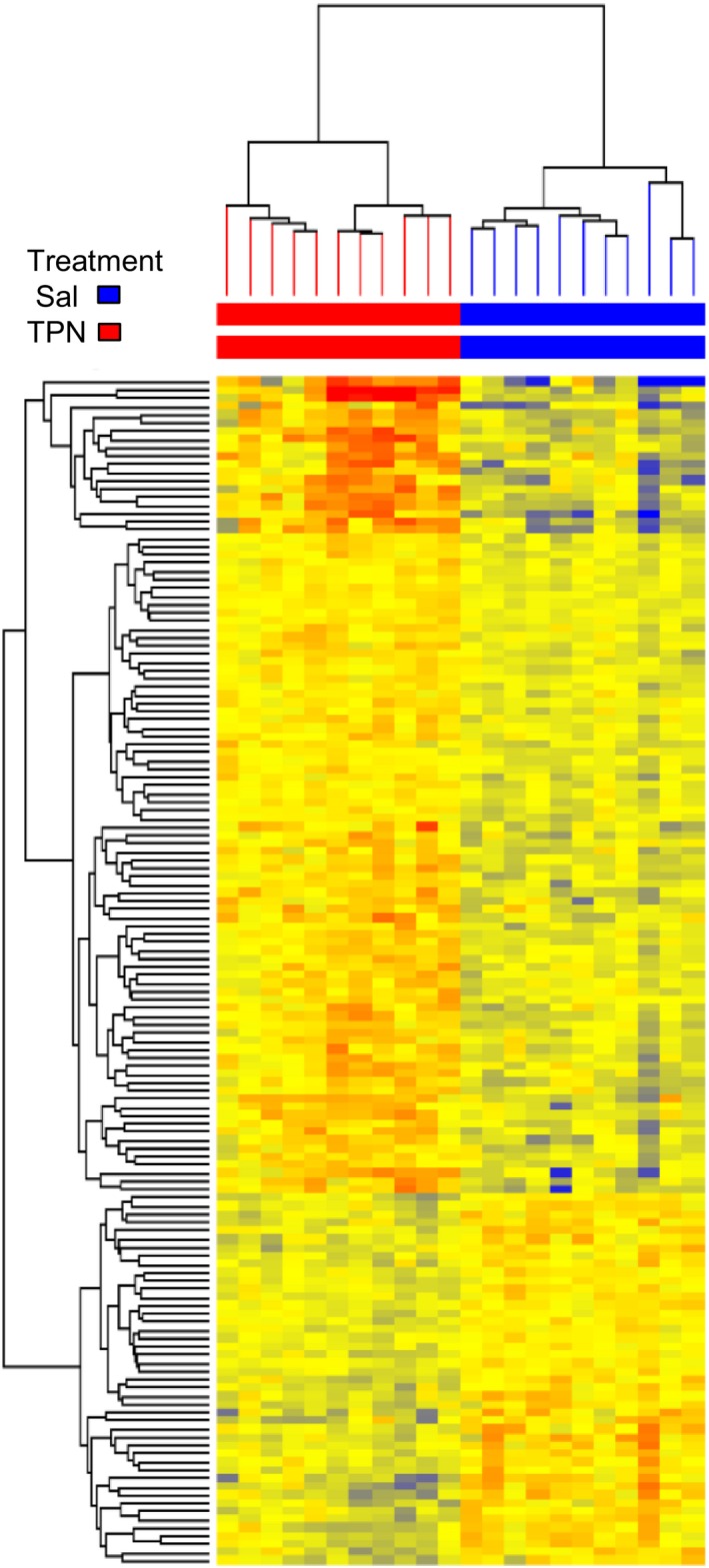
Cluster analysis of mRNAs with *P* < 0.05 in muscle biopsies from TPN‐treated patients and controls. All samples within one treatment, TPN or saline, clustered together.

137 transcripts remained differently expressed between the patient groups after statistical evaluation (*P* < 0.05) and 447 transcripts at *P* < 0.1. Of these, 177 transcripts were elevated and 270 showed lower expression in tissue from TPN‐treated patients compared to saline‐infused control patients. Cluster analysis showed that all samples within one treatment group clustered together (performed on transcripts significant at *P* < 0.05) (Fig. 1). A significance level of *P* < 0.10 (447 altered transcripts) were chosen to identify potentially altered gene transcripts for subsequent inclusion to algorithm analyses. A Gene ontology search was performed and indicated associations of altered genes with “general” biological processes such as immune response and cellular metabolic processes. This indicate that nutrition supply influenced a large number of cellular processes. A pathway search found a significant association with wikipathways “processing of capped intron containing pre‐mRNA” and “metabolism of amino acids and derivates”. By using the NLP (natural language processing) algorithms in the Genespring software, interactions between RPS6K1, ULK, and MAP kinase signaling was found. Selected transcripts related to synthesis and degradation of proteins are shown in Table 4, 5, 6.

**Table 4:** mRNA transcripts related to mTOR signaling, protein synthesis and autophagy/lysosomal degradation of proteins with significantly altered expression in muscle tissue from patients receiving overnight infusion of TPN at constant rate compared to saline‐infused control patients

Fold change	*P* (corr)	Gene symbol	Gene name	Entrez gene ID	Function
1.5	0.080	EIF4E2	Eukaryotic translation initiation factor 4E family member 2	9470	Translation initiation
1.3	0.094	EIF4A1	Eukaryotic translation initiation factor 4A1	1973	Translation initiation
1.3	0.097	EIF5	Eukaryotic translation initiation factor 5	1983	Translation initiation
1.3	0.097	GARS	Glycyl‐tRNA synthetase	2617	tRNA ligase
1.3	0.031	RPS6KB1	Ribosomal protein S6 kinase, 70 kDa, polypeptide 1	6198	Stimulation of protein synthesis and ribosome biogenesis,
1.3	0.065	MAPKAP1	Mitogen‐activated protein kinase‐associated protein 1 (also known as SIN 1)	79109	Member of mTORC2
1.3	0.049	KIAA0226	KIAA0226 (also known as Rubicon)	9711	Negative regulator of autophagy
1.2	0.049	CD164	CD164 molecule, sialomucin	8763	Protein targeted to lysosomes, implicated in myoblast fusion
−2.1	0.010	CTSC	Cathepsin C	1075	Lysosomal enzyme
−1.8	0.049	BMF	Bcl2 modifying factor	90427	Involved in apoptosis and autophagy
−1.7	0.004	ABHD16A	Abhydrolase domain containing 16A (NG26, BAT5)	7920	Protein reported as possibly associated with Rictor
−1.5	0.034	ULK1	unc‐51‐like kinase 1 (C. elegans)	8408	Autophagy initiation
−1.5	0.018	BCL2L11	BCL2 like 11 (also known as BIM)	10018	Involved in apoptosis and autophagy
−1.4	0.087	TFEB	Transcription factor EB	7942	Transcription factor for lysosomal genes
−1.4	0.098	DIRC2	Disrupted in renal carcinoma 2	84925	Lysosomal membrane protein
−1.2	0.096	ZFYVE1	Zinc finger, FYVE domain containing 1 (DFCP1)	53349	Possible role in autophagosome formation
−1.2	0.073	RIN2	Ras and Rab interactor 2	54453	Endocytic membrane traffic

**Table 5:** mRNA transcripts related to receptor and intracellular signaling with significantly altered expression in muscle tissue from patients receiving overnight infusion of TPN at constant rate compared to saline‐infused control patients

Fold change	*P* (corr)	Gene symbol	Gene name	Entrez gene ID
1.9	0.023	INPP4B	inositol polyphosphate‐4‐phosphatase, type II, 105 kDa	8821
1.5	0.097	PRKCE	protein kinase C, epsilon	5581
1.3	0.033	MAP2K4	mitogen‐activated protein kinase kinase 4	6416
1.3	0.094	IPPK	inositol 1,3,4,5,6‐pentakisphosphate 2‐kinase	64768
−2.4	0.006	PIK3IP1	phosphoinositide‐3‐kinase interacting protein 1	8501
−2.3	0.003	MOB3B	MOB kinase activator 3B	79817
−2.3	0.007	SLC43A1	solute carrier family 43 (amino acid system L transporter), member 1	113791
−1.5	0.098	IRS1	insulin receptor substrate 1	3667
−1.5	0.003	PLEKHM3	pleckstrin homology domain containing, family M, member 3	389072
−1.4	0.049	IGF1R	insulin‐like growth factor 1 receptor	3480
−1.4	0.057	ACVR2B	activin A receptor, type IIB	93
−1.3	0.060	PRKCQ	protein kinase C, theta	5588
−1.2	0.067	MAP2K7	mitogen‐activated protein kinase kinase 7	5609

**Table 6:** mRNA transcripts related to ubiquitin and proteasome degradation of proteins, with significantly altered expression in muscle tissue from patients receiving overnight infusion of TPN at constant rate compared to saline‐infused control patients

Fold change	*P* (corr)	Gene symbol	Gene name	Entrez gene ID
1.6	0.097	FBXO3	F‐box protein 3	26273
1.6	0.097	USP28	ubiquitin‐specific peptidase 28	57646
1.2	0.095	UBAP2L	ubiquitin‐associated protein 2‐like	9898
−1.8	0.097	FBXO32	F‐box protein 32	114907
−1.6	0.062	UBL4B	ubiquitin‐like 4B	164153
−1.5	0.020	FBXL20	F‐box and leucine‐rich repeat protein 20	84961
−1.4	0.057	UBE4A	ubiquitination factor E4A	9354
−1.3	0.078	UBE2G1	ubiquitin‐conjugating enzyme E2G 1	7326
−1.3	0.044	FBXO46	F‐box protein 46	23403
−1.2	0.098	USP3	ubiquitin‐specific peptidase 3	9960

#### Transcript alterations of genes related to mTOR signaling and translation initiation

**Figure 2:**
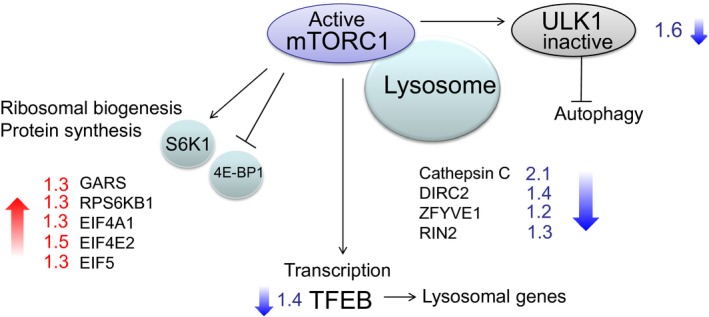
Fold changes in expression of transcripts related to mTOR signaling in muscle biopsies from patients who received TPN versus saline‐infused control patients. Active mTORC1 is attached to lysosomal membranes. Cells can “switch” between synthesis and degradation of proteins by alternating signals to mTORC1 and ULK 1. Genes related to protein synthesis were upregulated, whereas genes related to lysosomal degradation of proteins were downregulated in patients receiving TPN infusion compared to saline‐infused patients (*P* < 0.1).

Several mRNA transcripts of genes associated with mTOR, or being targets for signaling through mTOR (mechanistic target of rapamycin), were significantly different between TPN‐treated patients and control patients. The downstream mTORC1 (mTOR complex1) substrate S6K1, which is involved in stimulation of protein synthesis and ribosomal biogenesis, were increased in TPN‐treated patients compared to control patients (RPS6K1, up 1.3‐fold, *P* < 0.05). MAPKAP1, a component of mTORC2 (also called SIN 1) was also increased in TPN‐treated patients compared to saline‐infused controls (up 1.3‐fold, *P* < 0.1). The autophagy initiator unc‐51‐like kinase 1 (ULK‐1) and transcription factor EB (TFEB), which is involved in transcription of lysosomal genes were downregulated (−1.5‐ and −1.4‐fold, respectively (*P* < 0.5–0.1). Both ULK1 and TFEB are targets for mTORC1 signaling (Fig. 2, Table 4). In addition, several mRNA transcripts coding for translation initiation factors and transcripts related to ribosomal biogenesis, eIF4A1, eIF4E2, eIF5, and glycyl‐tRNA synthetase (up 1.3,1.5, 1.3, 1.3) were increased in TPN‐infused patients compared to saline‐infused patients (Fig. 2,Table 4, *P* <** **0.1).

#### Transcript levels of genes related to autophagy and lysosomal degradation of proteins

In addition to changes in ULK 1 (autophagy initiator), several other genes with functions in lysosomal degradation showed significantly altered tissue concentrations. The lysosomal enzyme Cathepsin C, DIRC2, a lysosomal membrane transport protein, ZFYVE1, and RIN2 with possible roles in autophagosome formation and endocytic membrane traffic were all lower in TPN‐treated patients (−2.1, −1.4, −1.2, −1.3, respectively, *P* < 0.01–0.1). KIAA0226 (also called Rubicon) function as a negative regulator of autophagy and were subsequently increased (up 1.3‐fold, *P* < 0.05) (Matsunaga et al. 2009). In addition, Bcl2 modifying factor (BMF) and BCL2 like 11 (BCL2L11 also known as BIM) displayed decreased mRNA levels in TPN‐treated patients (−1.8, −1.5, respectively, *P* < 0.05). Both BMF and BCL2L11 were recently reported to have a role in autophagic processes besides more known functions in apoptotic processes (Luo et al. 2012; Contreras et al. 2013). Moreover, CD164 was 1.3‐fold higher in TPN‐treated patients compared to control patients (*P* < 0.05). CD 164 (also called Sialomucin) is a lysosomal membrane protein reported to promote myogenesis by myoblast fusion into myotubes (Bae et al. 2008) (Table 4).

#### Transcripts for intracellular signaling and proteasome degradation of proteins

A number of mRNA transcripts of genes related to intracellular signaling and ubiquitin‐mediated degradation of proteins were significantly altered but did not show a consistent pattern of either up or downregulation (*P* < 0.1). Selected transcripts related to intracellular signaling are shown in Table 5. mRNAs related to ubiquitin and proteasome degradation of proteins are provided in Table 6 (*P* < 0.01–0.1).

## Discussion

Previous investigations on long‐term overall effects by artificial nutrition to induce improved metabolic status in undernourished patients have mainly been based on anthropometric and body compositional analyses (Martin et al. 2015), whereas more short‐term effects by nutrition are usually demonstrated by bolus or constant infusions of labeled amino acids to monitor whole body flux of substrates and amino acids across organs, although, such studies are few based on direct tissue measurements (Emery et al. 1984; Dillon et al. 2007). More recently, various physical and functional evaluations have also been used to confirm important outcome by nutritional support (Fouladiun et al. 2005, 2007). However, outcome results have been rather variable in the literature to suggest that nutritional support to hospitalized patients is either ineffective or only moderately efficient depending on a large number of different explanations. Our own studies have indicated that palliative TPN to cancer patients may prolong survival for a period, related to improved metabolism including skeletal muscle tissue (Lundholm et al. 2004). Whole body tyrosine flux in weight‐losing cancer patients suggested, that main effects by TPN was to attenuate net protein breakdown, whereas induction of protein resynthesis was low or even lacking in response to standard TPN regimens (Hyltander et al. 1991). In contrast, studies on weight stable and rather healthy individuals indicated possibilities to create net uptake of amino acids across skeletal muscle beds, pending high infusion rates that are usually not possible to apply in standard TPN regimens due to appearing side effects by some amino acids (Lundholm et al. 1987). Discrepant results among reported studies are probably not related to oral or intravenous routes for administration of nutrition solutions (Bennegard et al. 1984). Thus, it seems complex to correctly interpret effects by various nutrition regimens and it appears necessary to apply more direct methods for in vivo measurements to understand overall and net effects by different regimens on the complex patterns of protein metabolism in tissue compartments such as skeletal muscles, which is important for recovery and rehabilitation of patients on demanding treatment as cancer surgery and neoadjuvant oncological programs. Therefore, we found it appealing to apply algorithmic transcript analyses related to protein synthesis, protein degradation and related signaling in skeletal muscles cells to continue our work to improve nutritional support to hospitalized patients.

mTORC1 is central in a complex nutrient signaling pathway which integrates a network of signals from amino acids, growth factors and cellular energy state to induce or inhibit protein synthesis (Weigl 2012). It was early recognized that amino acids are necessary to activate muscle protein synthesis (Preedy and Garlick 1986). Later studies found that amino acids were potent activators of mTORC1, which in turn phosphorylates the downstream mTORC1 substrates 4E‐Binding protein 1 (4EBP1) and S6kinase1 (S6K1) to stimulate cap‐dependent translation and promote ribosomal biogenesis (Hara et al. 1998; Drummond et al. 2008). Our previous studies in healthy subjects indicated that the stimulation of protein synthesis seems to be related to the blood kinetics of infused amino acids (Lundholm et al. 1987). We have reported that both intensive care patients (ICU), as well as weight‐losing cancer patients, had low amino acid concentrations in plasma compared to overnight fasted healthy individuals; and that infusion of a standard parenteral nutrition regimen was not sufficient to raise plasma concentrations of amino acids to levels comparable to postprandial levels after oral meal intake by healthy subjects (Hyltander et al. 1991; Iresjo et al. 2006). However, in a previous study, we demonstrated increased S6kinase 1 (S6K1) and 4EBP1 phosphorylation as well as altered translation initiation factor complex formation, indicative of increased protein synthesis despite subnormal plasma amino acid concentrations during TPN infusion to stressed patients (Iresjo et al. 2008). In this study we found that mRNA transcripts of S6K1 and several translation initiation factors as well as t‐RNA synthetase were increased in muscle biopsies from the TPN‐treated patients. These observations support that capacity for protein translation may increase during standard TPN infusion despite subnormal total amino acid concentrations in plasma compared to healthy subjects (Le Boucher et al. 1997; Pitkanen et al. 2003).

Leucine is considered particularly important for protein synthesis and we have earlier reported that leucine stimulates protein synthesis in human muscle biopsies (Lundholm and Schersten 1977). Despite plasma leucine concentrations did not rise during overnight parenteral infusion, intracellular leucine levels may have been higher than in plasma; perhaps in agreement with decreased mRNA concentrations of the amino acid transporter SCL43A1 (LAT3), which is proposed to export leucine out of muscle cells (Table 5) (Fukuhara et al. 2007). Bohe et al. reported that protein synthesis became refractory despite continuous delivery of amino acids which is biologically evident, since it is not possible to expand muscle mass continuously by eating only (Bohe et al. 2001). Therefore, some “stop induction” of protein balance must occur in the presence of continuously elevated blood substrate levels. Thus, transcription (transcript levels), as evaluated in this study, may not have stopped during 15–16 h infusion in the presence of anabolic signaling, since the tissue may not have reached “muscle full”.

mTOR signaling is reported to regulate autophagy by phosphorylation of the autophagy initiator protein ULK1 in addition to stimulate protein synthesis. Accordingly, the most apparent “pattern” of altered mRNAs in this study occurred for genes associated with autophagy and lysosomal degradation of proteins (Kim et al. 2011; Settembre et al. 2012) (Table 4, Fig. 2). These transcripts were consistently decreased in our patients on nutrition infusion indicating that TPN suppressed autophagy/lysosomal protein degradation. In addition, a negative regulator of autophagy (Rubicon) was increased in TPN‐infused patients. This further supports suppression of autophagy. We and others have earlier reported that lysosomal degradation is increased in muscle tissue of weight‐losing cancer patients despite decreased release of 3‐methylhistidine across their leg muscle beds, indicative of decreased myofibrillar protein breakdown (Lundholm et al. 1982). We have reported increased enzyme activities of lysosomal Cathepsin D and acid phosphatases in muscles tissue from patients with various types of cancer (Schersten and Lundholm 1972), while others reported increased Cathepsin B and L activities as well as increased L3CB‐II/I ratio in muscle tissue from esophageal cancer patients (Tardif et al. 2013). Some genes with well‐known functions in apoptotic processes also displayed altered expression. However*,* BCL2 like 11 (also called BIM), and Bcl2 modifying factor (also known as BMF), which are binding partners of BCL2 –a protein with a well‐known role in apoptosis, are reported to have functions in autophagy as well (Luo et al. 2012; Contreras et al. 2013). Both BMF and BIM were found to interact with Beclin1, a key protein in autophagy. Therefore, it is likely that decreased mRNA levels of BIM and BMF during TPN in our present patients reflect roles in autophagy rather than involvement in apoptosis; since there is no support for increased apoptosis in muscle tissue of cachectic patients (Tardif et al. 2013).

Several signaling pathways are known as upstream activators of mTORC1 and mTORC2. Our present findings indicate involvement of various pathways, including the IGF‐1/PI3kinase pathway which is well‐known in regulation of muscle cell growth and proliferation (Philippou et al. 2007). In this study we found downregulation of both IGF‐1 receptor and IRS‐1 (insulin receptor substrate 1) mRNAs by nutrition infusion. These findings are similar to our previous findings in muscle tissue from starved and freely fed mice where IGF‐1receptor mRNA expression were decreased in fed mice compared to starved mice simultaneously with activation of translation initiation and stimulation of protein synthesis (Iresjo et al. 2013). mTORC2 is in contrast to mTORC1 considered to respond to growth factors through PI3kinase‐AKT‐dependent signaling (Frias et al. 2006). Mechanisms for activation as well as downstream effects of mTORC2 activation are, however, less defined compared to mTORC1. SIN 1, a subunit of mTORC2 (also called MAPKAP1), displayed increased levels in our TPN‐treated patients. SIN 1 participates in phosphorylation of AKT at ^ser 473;^ a signaling event downstream of both IGF‐1receptor and insulin receptor stimulation (Frias et al. 2006). Thus, findings of altered mRNA levels related to IGF‐1 receptor/PI3kinase and mTORC2 signaling should be of importance for muscle protein balance in patients on TPN as indicated in this study.

Muscle protein degradation occurs through specific degradation by the ubiquitin‐proteasome system besides degradation of proteins by lysosomes. Increased ubiquitin‐mediated protein degradation seems to be important in patients with critical illness and sepsis, but not so in patients with cancer cachexia (Klaude et al. 2007; Stephens et al. 2010). Accordingly, we found no consistent pattern of alterations in ubiquitin‐proteasome‐related genes. However, decreased expression was found for the ubiquitin ligase FBXO32 (also known as MAFbx/Atrogin). Both mRNA and protein levels of FBXO32 have previously been reported to be increased in various conditions of muscle wasting (Bodine and Baehr 2014). Thus, it may be of relevance for nutrition that FBXO32 mRNA levels decreased significantly by TPN in our patients (Table 6).

In conclusion, microarray analyses provided information relevant for skeletal muscle protein metabolism during TPN infusion. mRNA alterations in response to preoperative overnight intravenous nutrition indicate that short‐term standard TPN is effective to induce skeletal muscle anabolism; suppressing autophagy/lysosomal degradation and stimulate initiation of protein translation by signaling through mTOR.

## Conflict of Interest

The authors declare they have no conflict of interests.
